# Disproportionality analysis of European safety reports on autoimmune and rheumatic diseases following COVID-19 vaccination

**DOI:** 10.1038/s41598-025-98313-4

**Published:** 2025-04-27

**Authors:** Federica Fraenza, Cecilia Cagnotta, Mario Gaio, Liberata Sportiello, Cristina Scavone, Annalisa Capuano, Ugo Trama

**Affiliations:** 1 Department of Experimental Medicine, University of Campania “Luigi Vanvitelli”, Naples, Italy; 2 Campania Regional Centre for Pharmacovigilance and Pharmacoepidemiology, Naples, Italy; 3 Regional Pharmaceutical Unit, Campania Region, Naples, Italy; 4 Department of Life Science, Health, and Health Professions, Link Campus University, Rome, Italy

**Keywords:** Pharmacovigilance, COVID-19, Vaccines, Safety, Rheumatic diseases, Immune-mediated diseases, Health care, Rheumatology, Risk factors, Signs and symptoms

## Abstract

The safety profile of COVID-19 vaccines is well-established, yet the widespread immunization campaign has led to an increase in reported cases of Immune-Mediated and Rheumatic Diseases (IMDRs). This study aimed to assess the reporting of Adverse Events Following Immunization (AEFIs) related to IMDRs after COVID-19 vaccination. We analyzed all individual case safety reports (ICSRs) related to COVID-19 vaccines authorized in the European Union (i.e., tozinameran, elasomeran, ChAdOx1-S NCoV-19, and Ad26.Cov2.S) registered in the EudraVigilance (EV) database from January 1, 2021, to October 23, 2023. Our analysis identified ICSRs with events indicative of IMDRs and conducted disproportionality analysis (i.e., Reporting Odds Ratio (ROR) with 95% CI) to examine the frequency of different IMDR types linked to each vaccine. In total, 45,352 ICSRs reported at least one AEFI associated with rheumatic or autoimmune conditions, with 54% of them implicating tozinameran as the suspected vaccine. More than half of the reported AEFIs were classified as serious, with approximately 45% remaining unresolved. The most frequently reported conditions were other immune-mediated diseases, followed by arthritis, vasculitis, systemic lupus erythematosus, and tendinopathies. Our disproportionality analysis suggested that mRNA vaccines may be more frequently associated with new autoimmune rheumatic diseases. Stratified analysis revealed significant associations for ChAd, particularly in vasculitis and tendinopathies, only when compared to Ad26.Cov2.S. Real-world pharmacovigilance data suggest that autoimmune and rheumatic diseases may be under-reported following COVID-19 vaccination, highlighting the need for further research to better understand the underlying mechanisms. The findings from this disproportionality analysis suggest the need for further studies to investigate these results in greater depth.

**Supplementary Information:**

The online version contains supplementary material available at 10.1038/s41598-025-98313-4.

## Introduction

The pandemic of Coronavirus disease 2019 (COVID-19), caused by severe acute respiratory syndrome coronavirus 2 (SARS-CoV‐2), has been a global health emergency, as declared by the World Health Organization (WHO), that provoked more than seven million deaths worldwide from the declaration of pandemic to January 19, 2025^1–5^. Starting from 2020, vaccines based on different technologies have been developed in various countries to provide acquired immunity necessary to prevent the disease. The first COVID-19 vaccines to be authorized for emergency use in the European Union (EU) were: tozinameran (Pfizer-BioNTech – authorized in December 2020) and elasomeran (Moderna – authorized in January 2021), both mRNA-based vaccines; ChAdOx1-S NCoV-19 (AstraZeneca – authorized in January 2021), referred to as ChAd, and Ad26.Cov2.S (Janssen – authorized in March 2021) as viral vector vaccines^6–9^. mRNA vaccines contain genetic instructions for producing the spike protein of SARS-CoV-2, whereas viral vector vaccines use a different virus modified to include the gene that encodes the spike protein.

On June 17, 2020, the European Commission presented the EU Vaccines Strategy to accelerate the development, production, and distribution of COVID-19 vaccines. Since then, the Commission has secured up to 4.2 billion doses of vaccines. Vaccine deliveries to EU countries steadily increased, and by August 2023, 84.8% of the adult population had received at least one dose of the vaccine.

^10^.

Despite the development of COVID-19 vaccines providing to be both effective and well-tolerated, ongoing attention is being given to better characterize potential adverse events, defined as “adverse events following immunization” (AEFIs)^11,12^. Specifically, the widespread immunization program and the concurrent use of multiple COVID-19 vaccines in the real-world context has revealed various safety issues, such as new-onset or flare-up of immune-mediated and rheumatic diseases (IMRDs)^13–17^.

IMRDs are a heterogeneous group of inflammatory diseases affecting mainly musculoskeletal and connective tissue systems. The most common clinical signs of IMRDs include joint aches, swelling, redness over the joints, fatigue, and mild fever. Pathophysiological mechanisms involve self-reactive, antigen-driven immune responses, whose onset may result from interaction between genetic and environmental factors, as well as from hormonal alterations, infections, and viral vaccination^18,19^. Currently, the causal association between vaccination other than COVID-19 vaccines and autoimmunity is a topic of debate. Different data suggest a potential relation between vaccination (including vaccination against tetanus, rubella, hepatitis B) and IMRDs, such as the appearance of Guillain–Barré syndrome or inflammatory conditions, as arthritis or vasculitis^20,21^.

Similarly, COVID-19 vaccines may trigger autoimmune reactions as documented in literature by various case series and systematic reviews^21–24^.According to the literature, it seems that several.

reports of immune-mediated inflammatory diseases following COVID-19 vaccination have occurred during 4–8 weeks after the vaccine^25–28^. It has been proposed that various immunological processes, such as bystander activation (non-specific) and molecular mimicry (antigen-specific), may be linked to the development of autoimmune diseases or syndromes resembling autoinflammatory reactions following vaccinations^29^. Molecular mimicry occurs when foreign antigens resemble host proteins, triggering autoimmune responses. This mechanism, along with infection or a strong adjuvant, may contribute to post-vaccination autoimmunity. Bystander activation involves non-specific stimulation of dormant autoreactive T-cells (mainly CD8+) via cytokines like IL-15 and interferons. This immune overstimulation can result from infection or vaccination, but its full impact remains unclear and requires further research^22^.

Considering that the risk of IMRDs related to COVID-19 vaccines is not well understood, the analyses conducted on pharmacovigilance databases can be useful to collect new data and add new evidence to that obtained from clinical trials and observational studies. Therefore, the purpose of this study was to assess the reporting of AEFIs associated to IMRDs following COVID-19 vaccines by analyzing data from EudraVigilance (EV), the European Pharmacovigilance database, and to compare the reporting probability of these AEFIs for each of the COVID-19 vaccines.

## Materials and methods

### Data source

Data on Individual Case Safety Reports (ICSRs) related to COVID-19 vaccines authorized in the European Union – tozinameran, elasomeran, ChAd, and Ad26.Cov2.S – were retrieved from the EV database^30^. Although the ChAd vaccine has been withdrawn, we included it as it was widely administered in Europe, making its safety profile still relevant^31^. The EV contains all ICSRs reported by a healthcare professional or a consumer to a European Union national competent authority or a marketing authorization holder. Notably, ICSR reports from the UK are included in the EudraVigilance database, despite Brexit. In fact, following the UK’s departure from the EU on January 1, 2021, UK reports were categorized as non-EEA cases^32^. These data are publicly available for transparency through the EMA website (www.adrreports.eu). We selected all ICSRs reported in EV from 01/01/2021 to 23/10/2023.

### Study population

We selected all ICSRs describing AEFIs suggestive of rheumatic and immune-mediated diseases. To achieve this, we identified five Standardized MedDRA Queries (SMQs): “Arthritis,” “Tendinopathies,” “Vasculitis,” “Other immune-mediated disorders,” and “Systemic lupus erythematosus”, according to the Medical Dictionary for Regulatory Activities (MedDRA) dictionary^33^. The SMQ, a standardized terminology utilized for medical information classification, comprises a list of Preferred Terms (PTs) within MedDRA. Each PT is a distinct medical concept for a symptom, sign, disease diagnosis, therapeutic indication, investigation, surgical or medical procedure, and medical social or family anamnesis. We selected PTs aligned with our research question, and in cases where they were included in multiple SMQs, we grouped them into a single category with the following priority order: “Arthritis”, “Tendinopathies”, “Vasculitis”, “Systemic lupus erythematosus”, “Other immune-mediated disorders”. The complete list of PTs included in our analysis, along with their categorization into SMQs, is available in the Table S1. After deduplication, in order to obtain comparable datasets, we classified the ICSRs into four cohorts based on the suspected vaccines (considering only vaccines authorized by the European Medicines Agency until June 2021): tozinameran, ChAd, elasomeran, and Ad26.Cov2.S. Additionally, a fifth cohort was established to encompass cases of heterologous vaccination, categorized as “Mix”.

### Descriptive analysis

We conducted a descriptive analysis of the demographic characteristics of ICSRs and the key features of the suspected AEFIs, stratifying the results by each vaccine. Specifically, the following information were described: the report type (spontaneous or non-spontaneous), the gateway receipt date (i.e., the date of receipt of the ICSR in EudraVigilance), the primary source qualification (healthcare professional or consumer), the patients’ age group and sex, and all reported suspected AEFIs including their duration, outcome, and seriousness. Although there are no specific fields for comorbidities or pre-existing conditions in the ICSRs, we can use the information on suspected/concomitant medications as a proxy to identify such conditions. Moreover, the ICSRs include data on suspected and concomitant medications. A suspected drug is characterized as a medication potentially linked to the observed suspected adverse drug reaction (ADR), whereas a concomitant medication refers to a drug the patient is exposed to, which may not necessarily be associated with the suspected ADR. For each one, administration route, therapy duration, dosages, and therapeutic indication were reported. The frequencies of AEFIs by SMQ, their seriousness and outcomes were described as well. Regarding the seriousness, an event is defined as serious when the ADR results in death, is life-threatening, requires/prolongs hospitalization, results in persistent or significant disability/incapacity, is a congenital anomaly/birth defect, or results in some other clinically important condition^34^. The outcomes may be labelled as “Recovered/Resolved”, “Recovering/Resolving”, “Recovered/Resolved with Sequelae”, “Not Recovered/Not Resolved”, “Fatal”, and “Unknown”.

### Disproportionality analysis

We conducted a disproportionality analysis to evaluate the reporting frequency of the five SMQs associated with each vaccine. The analysis involved computing the Reporting Odds Ratio (ROR) along with a 95% confidence interval (95% CI). The ROR was determined as the ratio of the odds of cases reported with a specific COVID-19 vaccine compared to cases reported with another COVID-19 vaccine. Specifically, the ROR was calculated as (a/b)/(c/d), where ‘a’ represents the number of cases with PTs included in a specific SMQ with the selected vaccine, ‘b’ denotes the number of cases with PTs not included in that specific SMQ with the selected vaccine, ‘c’ signifies the number of cases with PTs included in a specific SMQ with the compared vaccine, and ‘d’ indicates the number of cases with PTs not included in that specific SMQ with the compared vaccine. The ROR was combined with an estimator of the statistical association, a Chi-squared test at one degree of freedom with Yate’s correction. We considered statistically significant an association with a ROR of at least two (with a lower limit of the 95% Confidence Interval (95% CI) > 1) and Chi-squared test of at least four (threshold value at one degree of freedom with *p*< 0.05 in a 2 × 2 table). These criteria are widely validated in previous pharmacovigilance studies for evaluating drug safety signal^35^. Furthermore, we performed a disproportionality analysis exclusively using ICSRs reported by healthcare professionals to assess whether the results remain consistent when limited to reports from healthcare professionals only^36^.

Finally, we conducted a subgroup analysis, considering only cases with or without an underlying rheumatic disease. We identified ICSRs pertaining to patients with pre-existing rheumatic diseases by selecting those ICSRs which included among the suspected or concomitant medications an antirheumatic drug (the list of drugs used is provided in the Table S2). So, we used the suspected/concomitant medications as a proxy for underlying disease in order to distinguish patients with newly onset disease from those experiencing relapse/exacerbation. The subgroup analysis was performed only for the SMQs whose PTs are most indicative of a rheumatic disease, (i.e., the PTs included in the SMQs “Arthritis”, “Vasculitis” and “Tendinopathies”).

Notably, the disproportionality analysis excluded the pediatric population (defined as the consumers aged between 0 and 17 years old) due to the approval of only two COVID-19 vaccine in this subgroup (i.e., tozinameran and elasomeran).

We followed the READUS-PV (Reporting of a Disproportionality Analysis for Drug Safety Signal Detection Using Individual Case Safety Reports in PharmacoVigilance) checklist as recommended for reporting findings from the disproportionality analysis^37^.

Data management and all statistical analysis were performed using the R Statistical Software (version 4.4.0; R Foundation for Statistical Computing, Wien, Austria).

## Results

**Fig. 1:**
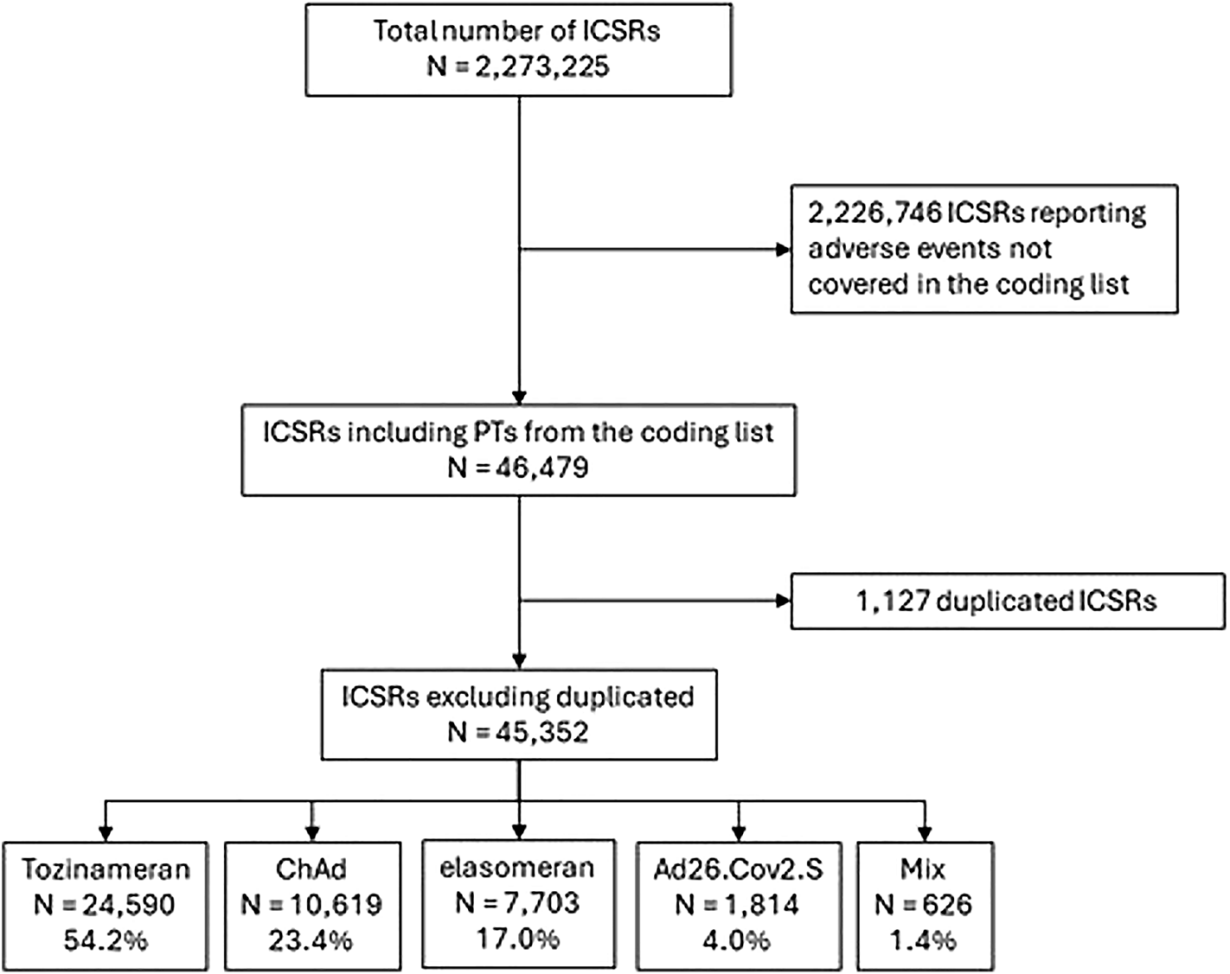
Flowchart for individual case safety reports (ICSRs) inclusion and exclusion criteria.

Between January 1, 2021, and October 23, 2023, a total of 2,273,225 ICSRs concerning COVID-19 vaccines were extracted from EV. Among these, 46,479 (2.0%) ICSRs reported at least one AEFI linked to immune-mediated and rheumatic diseases, as per the PTs listed in our coding list (See Table S1). After deduplication, 45,352 ICSRs were included in our analysis. Of these, over half reported tozinameran as the suspected vaccine (*N* = 24,590, 54.2%), followed by ChAd (*N* = 0,619, 23.4%), elasomeran (*N* = 7,703, 17.0%), and Ad26.Cov2.S (*N* = 1,814, 4.0%). Only 626 ICSRs (1.4%) indicated heterologous vaccination as the suspected vaccine (Fig. 1).

### Characteristics of the selected individual case safety reports (ICSRs)

Age was reported in 93% of the ICSRs. The most reported age group was related to adults (18–64 years) (65.7%), followed by elderly (65–85 years) (23.0%). The less reported group (2.2%) was of the pediatric patients (aged between 2 months and 17 years) and nearly 95% of their ICSRs identified tozinameran as the suspected vaccine. Sex was reported in over 98% of the cases, with female subjects being the most common in each of the five groups (i.e., reports including tozinameran, ChAd, elasomeran, Ad26.Cov2.S, and heterologous vaccination as suspected drug). All the ICSRs were spontaneously reported by healthcare professionals and consumers in equal proportions. However, Ad26.Cov2.S was reported as the suspected vaccine by healthcare professionals more frequently than by consumers, accounting for 61.2% of the reports. On average, the ICSRs included more than four adverse events, one suspected drug and almost two concomitant drugs other than the COVID-19 vaccine. Specifically, ICSRs including tozinameran, ChAd and elasomeran as suspected drug reported an average number of concomitant drugs of 1.53, 1.77, and 1.94, respectively; while ICSRs including Ad26.Cov2.S and heterologous vaccine as suspected drugs reported an average number of concomitant drugs of 2.00 and 2.12, respectively (Table 1).

**Table 1:** Characteristics of ICSRs included in the analysis.

	Tozinameran	ChAd	Elasomeran	Ad26.Cov2.S	Mix	Overall
N. of ICSRs	24,590	10,619	7,703	1,814	626	45,352
Age group (%)						
2 Months − 2 Years	4 (< 0.1)	0	2 (< 0.1)	0	0	6 (< 0.1)
3–11 Years	172 (0.7)	0	5 (0.1)	0	0	177 (0.4)
12–17 Years	711 (2.9)	0	42 (0.5)	0	3 (0.5)	756 (1.7)
18–64 Years	16,042 (65.2)	6,759 (63.7)	5,105 (66.3)	1,434 (79.1)	443 (70.8)	29,783 (65.7)
65–85 Years	5,700 (23.2)	2,337 (22.0)	1,991 (25.8)	252 (13.9)	136 (21.7)	10,416 (23.0)
More than 85 Years	311 (1.3)	553 (5.2)	59 (0.8)	9 (0.5)	2 (0.3)	934 (2.1)
Not Specified	1650 (6.7)	970 (9.1)	499 (6.5)	119 (6.6)	42 (6.7)	3280 (7.2)
Sex (%)						
Female	15,540 (63.2)	6342 (59.7)	4876 (63.3)	927 (51.1)	396 (63.3)	28,081 (61.9)
Male	8666 (35.2)	4008 (37.7)	2739 (35.6)	849 (46.8)	216 (34.5)	16,478 (36.3)
Not Specified	384 (1.6)	269 (2.5)	88 (1.1)	38 (2.1)	14 (2.2)	793 (1.7)
Report type = Spontaneous (%)	24,590 (100.0)	10,619 (100.0)	7703 (100.0)	1814 (100.0)	626 (100.0)	45,352 (100.0)
Primary source qualification = Consumer (%)	13,540 (55.1)	4694 (44.2)	3604 (46.8)	704 (38.8)	371 (59.3)	22,913 (50.5)
Mean n. of AEs per ICSR (± SD)	4.48 (5.47)	4.43 (5.59)	4.91 (5.63)	6.65 (7.47)	6.26 (8.07)	4.65 (5.68)
Mean n. of suspected drug per ICSR (± SD)	1.04 (0.31)	1.11 (0.92)	1.14 (0.52)	1.06 (0.29)	2.80 (4.48)	1.10 (0.79)
Mean n. of concomitant drug per ICSR (± SD)	1.53 (1.84)	1.77 (2.13)	1.94 (2.44)	2.00 (2.88)	2.12 (4.62)	1.68 (2.14)

### Characteristics of adverse events following immunization (AEFIs)

**Fig. 2:**
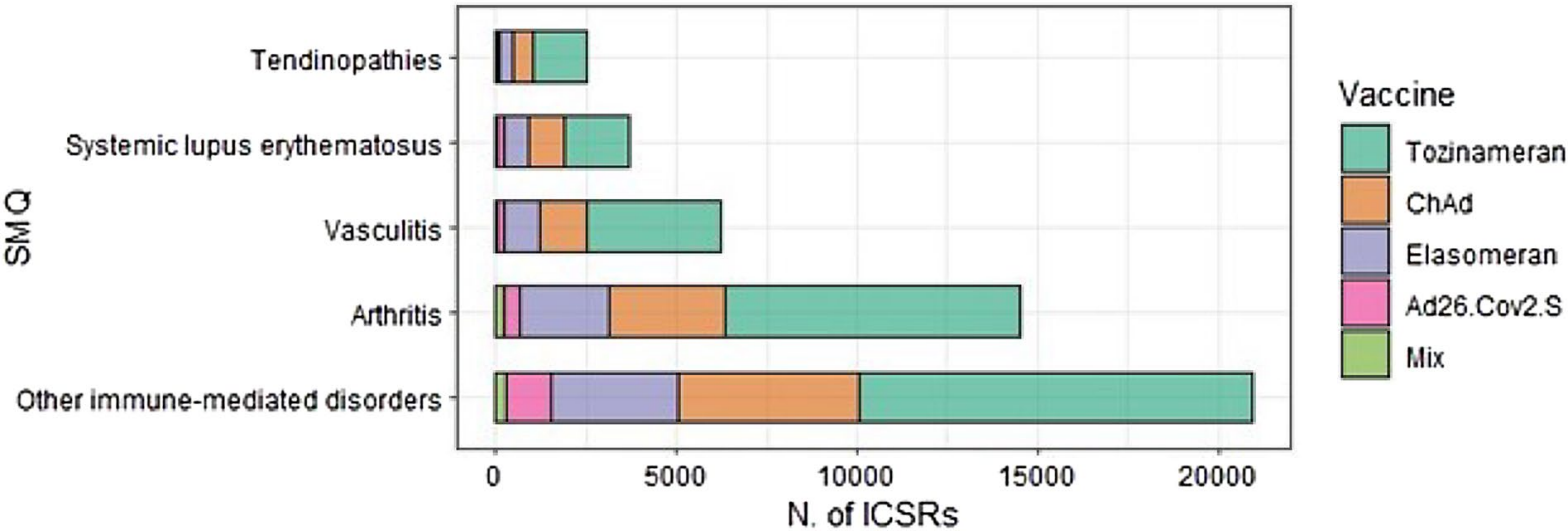
Distribution of adverse events following immunization (AEFIs) grouped as standardized MedDRA queries (SMQs), stratified by vaccine.

The most common Standardized MedDRA Query involved in the ICSRs included in the analysis was Other immune-mediated disorders (*N* = 20,949, 43.7%), followed by Arthritis (*N* = 14,518, 30.3%), Vasculitis (*N* = 6,240, 13.1%), Systemic lupus erythematosus (*N* = 3,668, 7.7%), and Tendinopathies (*N* = 2,509, 5.2%) (Fig. 2). Notably, the frequencies described above are numerically higher than the ICSRs. This is because each ICSR can encompass more than one adverse event.

**Fig. 3:**
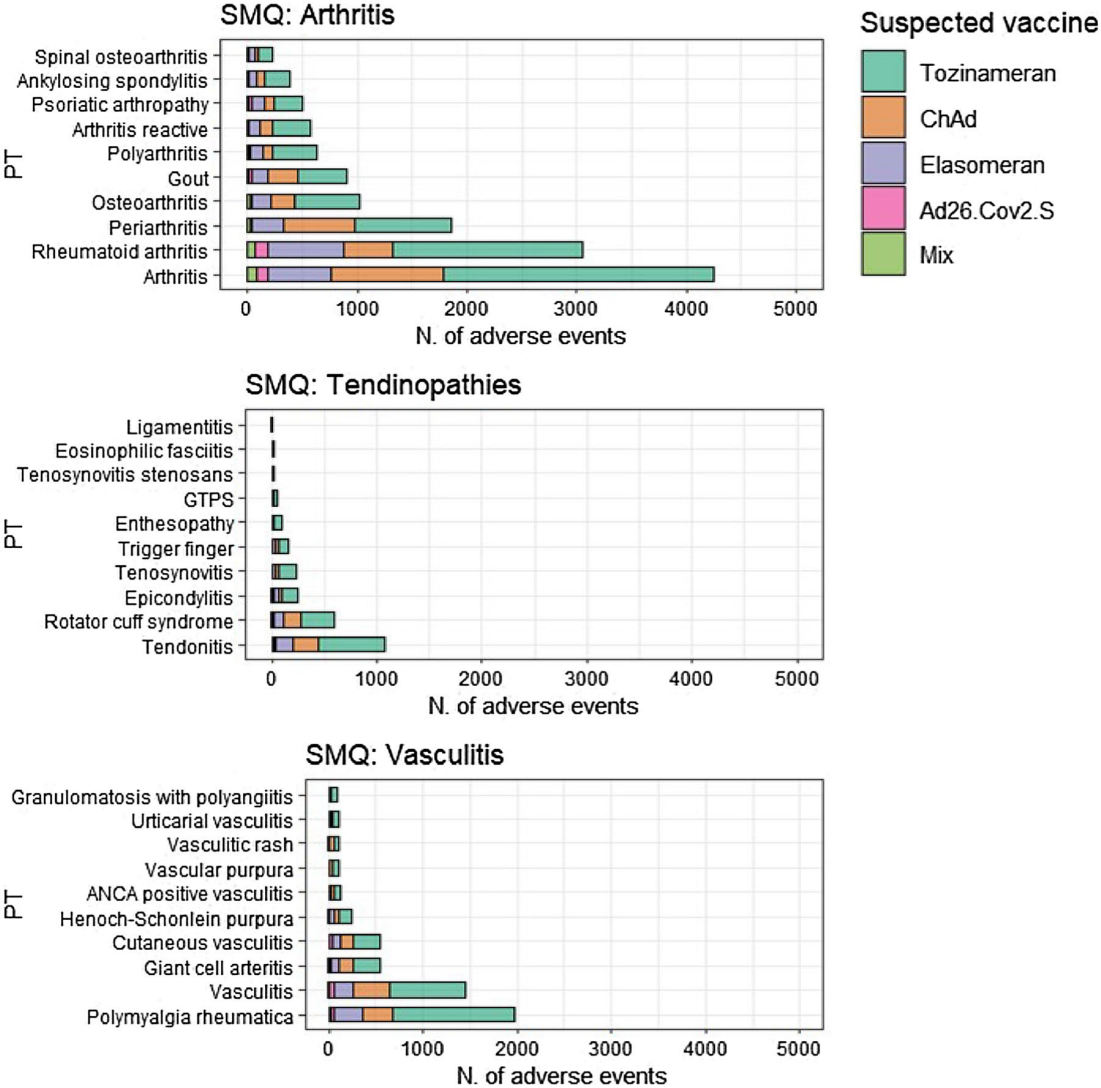
Distribution of adverse events following immunization (AEFIs) coded as Preferred Term (PT) by each Standardized MedDRA Query (SMQ) strictly related to rheumatic diseases (i.e., Arthritis, Tendinopathies, and Vasculitis), stratified by each vaccine.

**Fig. 4:**
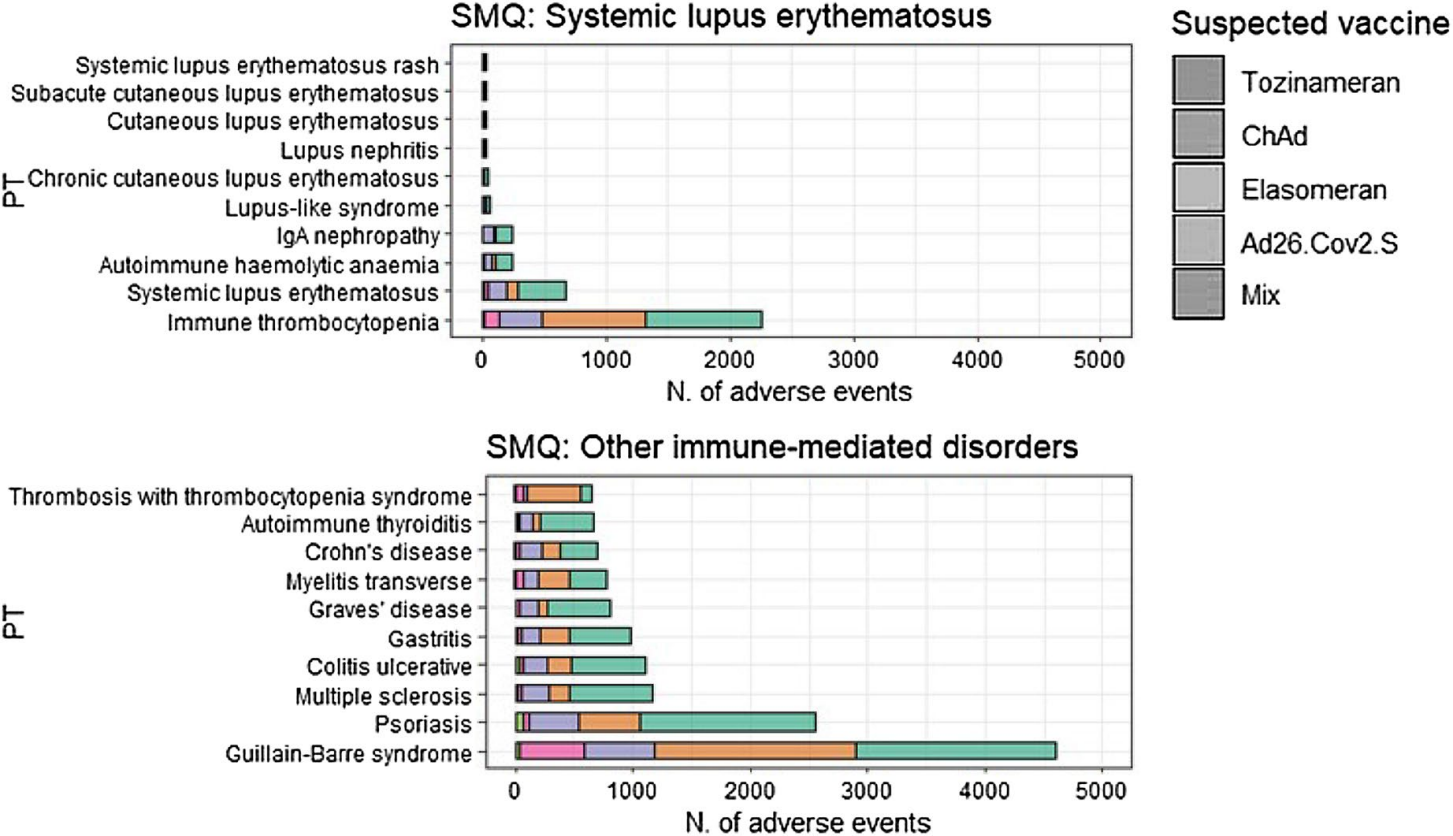
Distribution of adverse events following immunization (AEFIs) coded as Preferred Term (PT) by each Standardized MedDRA Query (SMQ) strictly related to immune-mediated diseases (i.e., Systemic lupus erythematosus, and Other immune-mediated disorders), stratified by each vaccine.

**Fig. 5:**
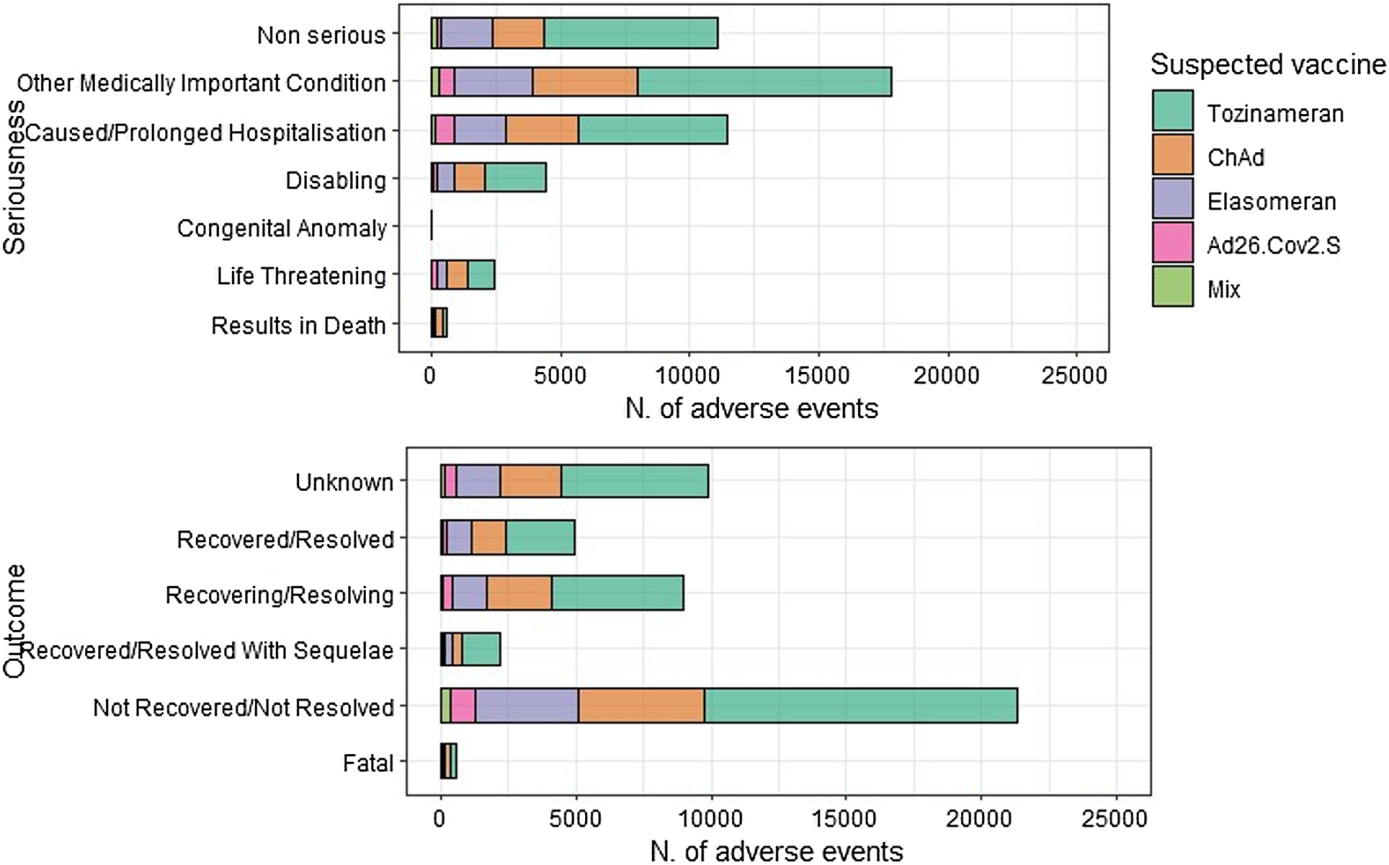
Distribution of Adverse Events Following Immunization (AEFIs) according to their seriousness and outcomes, stratified by vaccine.

In Figs. 3 and 4, we have expanded the SMQs to display the most commonly reported PTs for each SMQ, stratifying the results by the suspected vaccines. Within SMQ “Other immune-mediated disorders”, the most reported event was Guillan-Barrè syndrome (GBS) (*N* = 4,605), reported in 37.1% of cases following the administration of the tozinameran vaccine (*N* = 1,707), followed by ChAd (33.9%; *N* = 1,709), elasomeran (12.9%; *N* = 596), Ad26.Cov2.S (11.9%; *N* = 551) and heterologous vaccination (0.8%; *N* = 40). In the second most reported SMQ “Arthritis”, PT arthritis was the most reported event (*N* = 4,258), followed by rheumatoid arthritis (*N* = 3, 057). In particular, over half of the cases of arthritis (57.9%; *N* = 2,468) were reported following the administration of tozinameran vaccine, followed by ChAd (24.1%; *N* = 1,028), elasomeran (13.6%; *N* = 583), Ad26.Cov2.S (2.3%; *N* = 98) and heterologous vaccination (1.9%; *N* = 81). In the other SMQs of our interest, the most reported events were autoimmune thrombocytopenia included in SMQ “Sistemic Lupus Erythematous” (*N* = 2,250), polymyalgia rheumatica in SMQ “Vasculitis” (*N* = 1,966) and tendonitis in SMQ “Tendinopathies” (*N* = 1,080). More than half of serious AEFIs were classified as “Other medically important condition” (*N* = 17,798, 37.2%) or “Caused/prolonged hospitalization” (*N* = 11,484, 24.0%). 11,069 AEFIs (24.0%) were classified as “non serious”. The most common outcome was “Not recovered/not resolved” (*N* = 21,319, 44.5%). The most notable difference among the vaccines concerns the seriousness of AEFIs. Specifically, while non-serious AEFIs accounted for between 18% and 27% of the total reported AEFIs for tozinameran, ChAd, elasomeran, and mixed vaccinations, the frequency for Ad26.Cov2.S was 9.9%, suggesting a more frequent reporting of serious AEFIs with Ad26.Cov2.S. No relevant differences among the vaccines were observed regarding outcomes (Fig. 5).

### Disproportionality analysis

From the results of the disproportionality analysis, a lower likelihood of reporting rheumatic events from Ad26.Cov2.S compared to other vaccines was observed. Compared to Ad26.Cov2.S, the probability of reporting an AEFI in the SMQ “Arthritis” is higher following the administration of tozinameran (ROR = 2.46; 95% CI 2.21–2.73), ChAd (ROR = 2.25; 95% CI 2.02–2.50) and elasomeran (ROR = 2.16; 95% CI 1.93–2.41) vaccines. The same trend is observed in the SMQs “Vasculitis” and “Tendinophaties”. Furthermore, the Ad26.Cov2.S vaccine showed a clearly lower probability of reporting all three of the aforementioned SMQs compared to heterologous vaccination (in SMQ “Arthritis” ROR = 0.48; CI 95% 0.41–0.57, in SMQ “Vasculitis” ROR = 0.84; CI 95% 0.63–1.12, in SMQ “Tendinopathies” ROR = 0.37; CI 95% 0.24–0.58). No statistically significant associations were observed among the AEFIs related to the SMQs Systemic lupus erythematosus and Other immune-mediated disorders (Table 2). By analyzing only the cases reported by healthcare professionals, the associations related to the Ad26.Cov2.S vaccine were confirmed, including a lower likelihood of reporting arthritis compared to mixed vaccination, vasculitis with tozinameran, tendinopathies with all other vaccines, and other immune-mediated disorders with tozinameran, elasomeran, and mixed vaccination. However, the associations between IMRD events and other vaccines were not confirmed (Table S3).

**Table 2:** Disproportionality analysis grouped by each standardized MedRA query (SMQ).

SMQ	Vaccine	Comparator	Chi-squared	ROR (95% CI)
Arthritis	Tozinameran	ChAd	16.99	1.09 (1.05–1.14)
		Elasomeran	29.97	1.14 (1.09–1.19)
		Ad26.Cov2.S	**303.03**	**2.46 (2.21–2.73)**
		Mix	6.67	1.19 (1.04–1.36)
	ChAd	Elasomeran	2.14	1.04 (0.99–1.10)
		Ad26.Cov2.S	**226.30**	**2.25 (2.02–2.50)**
		Mix	1.48	1.08 (0.95–1.24)
	Elasomeran	Ad26.Cov2.S	**196.63**	**2.16 (1.93–2.41)**
		Mix	0.37	1.04 (0.91–1.20)
	Ad26.Cov2.S	Mix	**76.53**	**0.48 (0.41–0.57)**
Vasculitis	Tozinameran	ChAd	25.40	1.18 (1.10–1.25)
		Elasomeran	64.90	1.34 (1.25–1.44)
		Ad26.Cov2.S	**138.37**	**2.48 (2.12–2.90)**
		Mix	**34.31**	**2.08 (1.62–2.67)**
	ChAd	Elasomeran	9.54	1.14 (1.05–1.24)
		Ad26.Cov2.S	**84.50**	**2.11 (1.79–2.48)**
		Mix	19.68	1.77 (1.37–2.28)
	Elasomeran	Ad26.Cov2.S	53.60	1.84 (1.56–2.18)
		Mix	11.13	1.55 (1.20–2.00)
	Ad26.Cov2.S	Mix	1.21	0.84 (0.63–1.12)
Tendinopathies	Tozinameran	ChAd	15.43	1.22 (1.11–1.35)
		Elasomeran	22.17	1.30 (1.17–1.46)
		Ad26.Cov2.S	**88.00**	**3.88 (2.86–5.25)**
		Mix	4.59	1.44 (1.04–2.00)
	ChAd	Elasomeran	0.94	1.07 (0.94–1.22)
		Ad26.Cov2.S	**57.99**	**3.17 (2.32–4.33)**
		Mix	0.81	1.18 (0.84–1.65)
	Elasomeran	Ad26.Cov2.S	**49.48**	**2.96 (2.16–4.06)**
		Mix	0.25	1.10 (0.79–1.55)
	Ad26.Cov2.S	Mix	**19.64**	**0.37 (0.24–0.58)**
Systemic lupus erythematosus	Tozinameran	ChAd	71.07	0.71 (0.66–0.77)
		Elasomeran	9.23	0.87 (0.80–0.95)
		Ad26.Cov2.S	4.33	1.19 (1.01–1.40)
		Mix	8.70	1.63 (1.18–2.26)
	ChAd	Elasomeran	15.17	1.21 (1.10–1.34)
		Ad26.Cov2.S	35.88	1.66 (1.41–1.97)
		Mix	**25.55**	**2.28 (1.65–3.16)**
	Elasomeran	Ad26.Cov2.S	12.54	1.37 (1.15–1.63)
		Mix	14.17	1.88 (1.35–2.61)
	Ad26.Cov2.S	Mix	2.79	1.37 (0.96–1.96)
Other immune-mediated disorders	Tozinameran	ChAd	24.50	0.91 (0.88–0.95)
		Elasomeran	7.45	1.05 (1.01–1.10)
		Ad26.Cov2.S	3.05*10^−6^	1.00 (0.94–1.06)
		Mix	17.97	1.29 (1.15–1.46)
	ChAd	Elasomeran	39.30	1.15 (1.10–1.21)
		Ad26.Cov2.S	6.91	1.09 (1.02–1.17)
		Mix	31.77	1.41 (1.25–1.59)
	Elasomeran	Ad26.Cov2.S	2.39	0.94 (0.88–1.01)
		Mix	10.38	1.22 (1.08–1.38)
	Ad26.Cov2.S	Mix	14.53	1.29 (1.13–1.47)

Stratifying the disproportionality analysis by the presence/absence of underlying rheumatic diseases, the significant association for Ad26.Cov2.S resulted only in comparison with ChAd and only in vasculitis (ROR = 2.11; CI 95% 1.19–3.76) and tendinopathies (ROR = 6.28; CI 95% 1.53–25.84). On the contrary, in patients without underlying rheumatic disease, the associations observed in the overall analysis are confirmed and strengthened (Table 3).

**Table 3:** Disproportionality analysis grouped by each standardized MedRA query (SMQ) stratified by the presence/absence of underlying rheumatic diseases.

SMQ	Vaccine	Comparator	With underlying rheumatic diseases		Without underlying rheumatic diseases	
			Chi-quared	ROR (95% CI)	Chi-quared	ROR (95% CI)
Arthritis	Tozinameran	ChAd	57.92	0.59 (0.52–0.68)	30.79	1.13 (1.08–1.18)
		Elasomeran	0.03	1.02 (0.86–1.21)	22.97	1.12 (1.07–1.18)
		Ad26.Cov2.S	0.31	1.10 (0.82–1.46)	**302.56**	**2.59 (2.31–2.89)**
		Mix	0.11	0.90 (0.58–1.41)	6.57	1.19 (1.04–1.37)
	ChAd	Elasomeran	34.17	1.71 (1.43–2.05	0.07	0.99 (0.94–1.05)
		Ad26.Cov2.S	16.75	1.84 (1.37–2.47)	**208.56**	**2.28 (2.03–2.56)**
		Mix	2.98	1.52 (0.97–2.38)	0.53	1.05 (0.92–1.21)
	Elasomeran	Ad26.Cov2.S	0.14	1.07 (0.79–1.47)	**207.52**	**2.30 (2.04–2.58)**
		Mix	0.15	0.88 (0.55–1.41)	0.70	1.06 (0.92–1.22)
	Ad26.Cov2.S	Mix	0.36	0.82 (0.49–1.38)	**79.31**	**0.46 (0.39–0.55)**
Vasculitis	Tozinameran	ChAd	5.67	0.74 (0.59–0.94)	19.11	1.16 (1.08–1.24)
		Elasomeran	0.61	0.88 (0.68–1.17)	56.10	1.33 (1.24–1.44)
		Ad26.Cov2.S	2.15	1.57 (0.89–2.76)	**122.26**	**2.44 (2.07–2.87)**
		Mix	0.64	1.65 (0.61–4.44)	**29.04**	**2.01 (1.56–2.60)**
	ChAd	Elasomeran	1.19	1.19 (0.88–1.61)	9.75	1.15 (1.05–1.26)
		Ad26.Cov2.S	**6.20**	**2.11 (1.19–3.76)**	**77.41**	**2.10 (1.78–2.49)**
		Mix	2.02	2.21 (0.81–6.01)	17.16	1.73 (1.34–2.25)
	Elasomeran	Ad26.Cov2.S	3.20	1.77 (0.98–3.19)	47.75	1.83 (1.54–2.17)
		Mix	1.04	1.85 (0.68–5.08)	9.15	1.51 (1.16–1.96)
	Ad26.Cov2.S	Mix	2.22*10^−29^	1.04 (0.34–3.22)	1.36	0.83 (0.61–1.11)
Tendinopathies	Tozinameran	ChAd	5.24	0.65 (0.45–0.93)	20.96	1.28 (1.15–1.42)
		Elasomeran	1.04	1.34 (0.81–2.22)	20.92	1.31 (1.16–1.46)
		Ad26.Cov2.S	3.71	4.06 (0.99–16.54)	**82.79**	**3.85 (2.82–5.25)**
		Mix	0.01	1.31 (0.32–5.34)	4.35	1.45 (1.03–2.02)
	ChAd	Elasomeran	**6.94**	**2.07 (1.22–3.52)**	0.09	1.02 (0.89–1.17)
		Ad26.Cov2.S	**7.55**	**6.28 (1.53–25.84)**	49.82	3.01 (2.19–4.15)
		Mix	0.54	2.03 (0.49–8.34)	0.39	1.13 (0.80–1.60)
	Elasomeran	Ad26.Cov2.S	1.73	3.03 (0.70–13.01)	**46.44**	**2.94 (2.13–4.06)**
		Mix	2.23*10^−28^	0.98 (0.23–4.20)	0.24	1.11 (0.78–1.57)
	Ad26.Cov2.S	Mix	0.37	0.32 (0.04–2.29)	**18.25**	0.37 **(0.24–0.59)**

Ad26.Cov2.S is associated with a lower likelihood of reporting arthritis, vasculitis, and tendinopathies in patients without underlying rheumatic disease compared to those with pre-existing rheumatic disease. Therefore, for Ad26.Cov2.S, it seems more likely that a flare occurs in patients with pre-existing rheumatic disease rather than in those without. In contrast, the likelihood of reporting IMRD events such as arthritis, vasculitis, and tendinopathies with tozinameran, ChAd or elasomeran appears to be higher in patients without underlying rheumatic diseases.

## Discussion

To our knowledge, this is the first analysis of pharmacovigilance European data on rheumatic and immune-mediated adverse events following immunizations (AEFIs) following COVID-19 vaccination. As already documented by various case series and systematic reviews, COVID-19 vaccines may trigger autoimmune reactions. Specifically, fourteen diagnoses of immune-mediated and rheumatic diseases (IMRDs) (e.g., rheumatoid arthritis, vasculitis, and systemic lupus erythematosus) following COVID-19 vaccination (prevalently receiving ChAd) were observed in adult patients who were subsequently treated with disease-modifying antirheumatic drugs to manage their clinical manifestations^22^. In two case series, 66 and 39 patients had experienced transient IMRDs following COVID-19 vaccination^23,38^. The analyses revealed cases of polymyalgia rheumatica and arthritis. Furthermore, most of these subjects had received tozinameran and ChAd about ten days before the onset. Moreover, another study identified 24 cases of new onsets and 6 of flares of IMRDs following COVID-19 vaccination. Also in this study, most of the subjects received tozinameran and the most common manifestations were inflammatory arthritis and adult-onset Still’s disease^39^.

The present study aimed to evaluate the reporting frequency of immune-mediated and rheumatic diseases related to COVID-19 vaccines by using data retrieved from EudraVigilance (EV), the European Pharmacovigilance database. The EV contains all spontaneous or non-spontaneous reports, identified as ICSRs, of suspected adverse reactions to drugs or vaccines. Each ICSR must contain the following minimum data: (1) an identifiable reporter, (2) an identifiable patient, (3) an adverse event and (4) a suspect medicinal product (drug or vaccine)^40^. To provide a descriptive analysis of these events, we have retrieved over 2 million ICSRs, relating to the period from 1 January 2021 to 23 October 2023, and filtered for the ones that reported at last one AEFI related to a rheumatic and/or autoimmune disorder (*N* = 45,352).

Although the safety and efficacy profile of COVID-19 vaccines has been widely demonstrated, the mass vaccination campaign has led to an increased number of adverse events reported in the general population^41^. The most common adverse events were moderate and transient, while primarily included pain and redness at the injection site, myalgia, nausea, and hypersensitivity reactions^42^. However, in addition to these, an increasing number of cases of rheumatic and immune-mediated diseases have been reported in the literature^43^.

Looking at our data, only 2% of all ICSRs collected from EudraVigilance have at least one adverse event categorized as rheumatic or autoimmune disease, according to the selected Standardized MedDRA Queries. Therefore, rheumatic and autoimmune events are less reported than other adverse events following COVID-19 vaccinations. This result is in line with the literature, where these events are considered rare^22^.

According to our findings, the majority of the ICSRs including PTs related to rheumatic diseases were associated to an mRNA vaccine, especially to tozinameran (54.2%) that was the first vaccine authorized in the EU and widely used compared to other vaccines. However, it is important to note the lack of a denominator for spontaneously reported data. As such, direct comparisons of the number of events reported for each vaccine should be interpreted with caution, as the reporting rate may not be representative of the actual incidence of adverse events. Since this limitation affects all the vaccines under study, performing a disproportionality analysis would mitigate this issue, allowing for more robust comparisons across vaccines, as discussed below^44–47^.

As a result of the EMA’s vaccination campaigns in all Member States, which were primarily directed at adults and vulnerable patients, the majority of ICSRs in our dataset comprised the age groups 18–64 and 65–85^48^. Regarding to the sex distribution, we found an increased number of ICSR related to female patients. Firstly, data from the literature shows that women tend to have a higher risk of adverse drug reactions, and several studies emphasized that the number of safety reports is more in women than in men^49,50^. Moreover, according to the literature, there is a marked gender difference in the onset of autoimmune rheumatic diseases^51^. The causes are probably to be found in hormonal changes, induced not only by the intake of oral contraceptives, but also by physiological changes linked to the state of pregnancy or menstruation. Further causes could be related to genetic or environmental factors^51^.

Overall, half of the reporters were consumers. This is unusual with what usually happens in the spontaneous pharmacovigilance system, where healthcare professionals are around seven times more involved in reporting suspected ADRs than consumers^52^. However, during the COVID-19 vaccination campaign, there was a significantly higher participation of consumers compared to the usual trend. The high proportion of non-professional reports is a potential limitation of our analysis. In fact, while non-professional reports are valuable, their accuracy might differ, potentially influencing the results. To address this, we conducted a separate analysis focusing on healthcare professional reports, which confirmed key associations, as discussed below. However, the impact of non-professional reports on the overall findings should still be considered. While this data highlights the importance of patients as a source of safety information, in accordance with the new European Pharmacovigilance Legislation, it also raises concerns about the reporting’s reliability. On the other hand, non-professional reports have the potential to uncover unknown signals because they give multiple categories of suspected ADRs for different types of medications^53,54^. Specifically, consumers may be more inclined to report subjective symptoms such as pain, while HCPs might focus on more clinically severe or objective conditions. In fact, our data indicate that ICSRs submitted by healthcare professionals had a higher frequency of serious events, including death, life-threatening conditions, and hospitalization, whereas ICSRs submitted by consumers more frequently reported non-serious events (data not shown). Additionally, during the COVID-19 pandemic, HCPs were under significant pressure, which may have deprioritized the reporting of less severe events, leading to a higher proportion of reports coming from patients.

Our results indicated that among the events of our interest, Guillain Barré Syndrome (GBS) (9.6%), arthritis (8.9%), rheumatoid arthritis (6.4%), and psoriasis (5.3%) were the most often reported events (with a frequency > 5%). GBS is a rare autoimmune neurological disease that affects peripheral nerves and nerve roots. Recent evidence showed how the development of GBS is associated not only with vaccines for rabies, hepatitis A and B and influenza, but also with those against the COVID-19 disease^55–57^. Our data showed an increased likelihood of reporting GBS following administration of tozinameran and ChAd.

At the base, there would be a cross-reactive response of antibodies directed both against the spike protein containing in the inoculated vaccines and against gangliosides (peripheral nervous components), resulting in GBS. This phenomenon develops due to molecular similarity or “molecular mimicry” between the pathogen’s peptides that are included in the vaccine and self-antigens, with the formation of autoantibodies. Vaccination-induced autoimmunity could be further enhanced by the use of adjuvants, a variety of compounds used to stimulate the immune response, as well as by the genetic susceptibility of genetically predisposed individuals following exposure to vaccines^58^. However, assays for anti-ganglioside antibodies conducted on patients with post-vaccination GBS, reveal antibody levels significantly lower than those of other documented GBS cases, suggesting the possibility of other vaccine-related variables involved in the pathology’s genesis. In fact, in many cases of GBS reported in the literature there was a temporality between administration of the vaccine and onset of the pathology, strengthening the possible causal link. On the other hand, when considering a mass vaccination campaign, the incidence of this pathology in the population should also be evaluated, independently of any immunization plan. Several studies are currently underway to better understand the relationship between administration of the COVID-19 vaccines and the onset of GBS^24,59^.

Similarly, there are several cases reported in the literature of new onset arthritis post COVID-19 vaccination. These include cases of rheumatoid arthritis, vasculitis, systemic lupus erythematosus, and other immune-related disease^14,60–62^. Arthritis is an inflammatory disease characterized by inflammation and degeneration of the joints and surrounding tissues, with an etiology linked mainly to autoimmune reactions. Several clinical studies have demonstrated that vaccination against COVID-19 can induce a strong immune response. From our analysis, it emerges that the onset of arthritis mainly occurs following the administration of mRNA vaccines. According to Watanabe et al., the mRNA of the COVID-19 vaccines may induce an uncontrolled production of proinflammatory cytokines, responsible for immune system hyper-activation^63^. Two well-established factors in the etiopathogenesis of arthritis are tumor necrosis factor-alpha (TNF-α) and interleukin-6 (IL-6), two key pro-inflammatory cytokines involved in immune-mediated diseases. TNF-α plays a crucial role in promoting inflammation, synovial proliferation, and joint destruction, while IL-6 contributes to the differentiation of pathogenic T cells and the production of autoantibodies. Both cytokines are overexpressed following post-vaccination immune stimulation, which may contribute to immune-related adverse events^64,65^. Less clear, however, is the role of INF-I, whose over-expression has been found in several cases of post-vaccination arthritis^66^. It was hypothesized that type INF-I, stimulated by the vaccine mRNA, could act upstream of the cytokine cascade and, in turn, induce the over-production of IL-6 and TNF-a directly involved in the development of arthritis^63^. From a systematic review, it emerged that following appropriate treatment based on corticosteroids, the symptoms regressed or disappeared, suggesting that arthritic symptoms may be a transient response to the vaccine^67^.

According to the literature, our disproportionality analysis shows that the reporting frequency of newly diagnosed autoimmune rheumatic diseases seems to be higher for COVID-19 mRNA vaccines, with a homologous schedule, versus vector vaccines. Through the additional suspected/concomitant drugs reported in the ICSRs it was possible to stratify the disproportionality analysis based on the supposed presence/absence of underlying rheumatic diseases. As expected, the reporting of autoimmune rheumatic diseases related to COVID-19 vaccines was less frequent in cases already showing an underlying rheumatic pathology, except for vasculitis and tendinopathies, strengthening a more attention in the reporting the COVID-19 vaccines as suspect medicinal products in the occurrence of newly diagnosed autoimmune rheumatic diseases.

One of the main strengths of our study is its use of data retrieved from one of the largest collections of spontaneous reports^30^. This dataset encompasses diverse information from various countries and populations and is commonly utilized to identify potential risks and emerging concerns. Indeed, the core purpose of pharmacovigilance lies in addressing the limitations of pre-marketing clinical trials, which often involve small sample sizes, short study durations, and the exclusion or underrepresentation of certain population groups^68,69^. However, it is important to note that our analysis primarily relies on data from European Union countries (including the United Kingdom). When comparing our results with other studies assessing adverse events globally, we observe that, like in our study, tozinameran is the most frequently reported COVID-19 vaccine suspected of causing IMRD events. Moreover, like our study, these studies found an association between mRNA-based vaccines and IMRD events while no such association was observed for viral vector vaccines^70,71^.

The results from our study are subject to typical pharmacovigilance biases, including under-reporting, the tendency for serious events to be reported more frequently than non-serious events, and notoriety bias (the spontaneous reporting may be influenced in unknown ways by safety alerts)^72^. Additionally, it is important to note that half of the ICSRs in our analysis were reported by consumers, which may have impacted the overall quality and completeness of the reports. In particular, the lack of data regarding the number of doses administered did not allow us to perform an analysis by dose. For the same reason, the lack of information regarding any previous vaccinations did not allow us to distinguish with certainty homologous vaccinations from heterologous ones. Furthermore, we relied on the ATC codes of suspected or concomitant drugs to identify and distinguish between patients with newly onset disease and those experiencing relapse or exacerbation. Naturally, not all cases of underlying rheumatic disease could be detected, as we did not expect to find comprehensive and detailed clinical information on the patients included in the ICSRs. In fact, confounding factors, such as comorbidities, concomitant medications, and also the stress associated with the pandemic period and the reduced physical activity, cannot be fully assessed using a pharmacovigilance approach. Since pre-existing conditions and comorbidities are not mandatory fields in the ICSR, there is a potential for misclassification in identifying underlying immune-related diseases. Finally, given the nature of the analyzed data and considering the lack of a denominator for spontaneously reported data, we cannot infer causality and further pharmacoepidemiologic studies are therefore required to investigate these findings in greater depth. In fact, in pharmacovigilance studies using spontaneously reported data, we can only observe the tip of the iceberg. We rely on reported events from all vaccinated individuals, without knowing the total number of vaccinated individuals or the number of individuals who experienced the events.

Data management and all statistical analysis were performed using the R Statistical Software (version 4.4.0; R Foundation for Statistical Computing, Wien, Austria).

## Conclusion

The present study provides an overview of spontaneous reports of autoimmune rheumatic adverse events in subjects vaccinated with COVID-19 vaccines approved until June 2021 (tozinameran, elasomeran, ChAd and Ad26.Cov2.S). The results of this post-marketing analysis suggest that in terms of spontaneous reporting COVID-19, IMRD events were reported at a higher frequency with mRNA vaccines compared with reports following a viral vector vaccine. However, in agreement with the available evidence on the rare occurrence of these events, they covered a little percentage of the all events reported following COVID-19 vaccination. Our findings contribute to the ongoing monitoring of vaccine safety by identifying potential associations between COVID-19 vaccines and IMRD. While spontaneous reporting databases cannot establish causality, they play a crucial role in detecting safety signals that warrant further investigation. These results underscore the importance of continuous pharmacovigilance, particularly for mRNA vaccines, and highlight the need for long-term follow-up studies to better understand the underlying mechanisms. Additionally, our study reinforces the value of stratified risk assessments in public health policies, ensuring that vaccine recommendations remain evidence-based while maintaining public confidence in immunization programs. Despite the fact that vaccinations are generally safe to deliver, healthcare professionals should be aware of the risk factors for rheumatic disorders that can develop after vaccination.

## Electronic supplementary material

Below is the link to the electronic supplementary material.

Supplementary Material 1

Supplementary Material 2

Supplementary Material 3

## Data Availability

The datasets used and analyzed during the current study are available from the corresponding author on reasonable request.
