# Supplementary material for: Disproportionality analysis of European safety reports on autoimmune and rheumatic diseases following COVID-19 vaccination
Source: Sci Rep. 2025 Apr 27;15:14740. doi: 10.1038/s41598-025-98313-4 (PMC12034749; doi:10.1038/s41598-025-98313-4)
Supplement: Supplementary file 1 — Supplementary Material 1 [file 41598_2025_98313_MOESM1_ESM.docx]

**Table S1.** List of adverse events included in the analysis coded as preferred term (PT) and grouped by standardized MedDRA query (SMQ).

| **Included Preferred Terms** | **Group** |
| --- | --- |
| Ankylosing spondylitis | Arthritis |
| Arthritis | Arthritis |
| Arthritis climacteric | Arthritis |
| Arthritis enteropathic | Arthritis |
| Arthritis reactive | Arthritis |
| Chondromalacia | Arthritis |
| Felty's syndrome | Arthritis |
| Gout | Arthritis |
| Gouty arthritis | Arthritis |
| Nodal osteoarthritis | Arthritis |
| Osteoarthritis | Arthritis |
| Periarthritis | Arthritis |
| Polyarthritis | Arthritis |
| Psoriatic arthropathy | Arthritis |
| Rheumatoid arthritis | Arthritis |
| Sacroiliitis | Arthritis |
| SLE arthritis | Arthritis |
| Spinal osteoarthritis | Arthritis |
| Still's disease | Arthritis |
| Synovitis | Arthritis |
| Temporomandibular joint syndrome | Arthritis |
| Facet joint syndrome | Arthritis |
| Juvenile idiopathic arthritis | Arthritis |
| Laryngeal rheumatoid arthritis | Arthritis |
| Spondylitis | Arthritis |
| Amyloid arthropathy | Arthritis |
| Periarthritis calcarea | Arthritis |
| Haemophilic arthropathy | Arthritis |
| Caplan's syndrome | Arthritis |
| Plica syndrome | Arthritis |
| Carcinomatous polyarthritis | Arthritis |
| Autoimmune arthritis | Arthritis |
| Axial spondyloarthritis | Arthritis |
| Rapidly progressive osteoarthritis | Arthritis |
| Enteropathic spondylitis | Arthritis |
| Juvenile psoriatic arthritis | Arthritis |
| Juvenile spondyloarthritis | Arthritis |
| Paraneoplastic arthritis | Arthritis |
| Diffuse idiopathic skeletal hyperostosis | Arthritis |
| Idiopathic condylar resorption | Arthritis |
| Oligoarthritis | Arthritis |
| Chondronecrosis | Arthritis |
| Immune-mediated arthritis | Arthritis |
| Hydroxyapatite crystal deposition disease | Arthritis |
| Acute aseptic arthritis | Arthritis |
| Peripheral spondyloarthritis | Arthritis |
| Undifferentiated spondyloarthritis | Arthritis |
| Yao syndrome | Arthritis |
| Paradoxical psoriatic arthritis | Arthritis |
| Palindromic rheumatism | Arthritis |
| Enthesopathy | Tendinopathies |
| Eosinophilic fasciitis | Tendinopathies |
| Epicondylitis | Tendinopathies |
| Fibrosis tendinous | Tendinopathies |
| Rotator cuff syndrome | Tendinopathies |
| Tendonitis | Tendinopathies |
| Tenosynovitis | Tendinopathies |
| Trigger finger | Tendinopathies |
| Sinus tarsi syndrome | Tendinopathies |
| Tenosynovitis stenosans | Tendinopathies |
| Syndesmophyte | Tendinopathies |
| Ligamentitis | Tendinopathies |
| Tendon necrosis | Tendinopathies |
| Pellegrini Stieda disease | Tendinopathies |
| Painful os peroneum syndrome | Tendinopathies |
| Intersection syndrome | Tendinopathies |
| Cuboid syndrome | Tendinopathies |
| Greater trochanteric pain syndrome | Tendinopathies |
| Aortitis | Vasculitis |
| Arteritis | Vasculitis |
| Arteritis coronary | Vasculitis |
| Behcet's syndrome | Vasculitis |
| Cerebral arteritis | Vasculitis |
| Cryoglobulinaemia | Vasculitis |
| Cutaneous vasculitis | Vasculitis |
| Diffuse vasculitis | Vasculitis |
| Erythema induratum | Vasculitis |
| Giant cell arteritis | Vasculitis |
| Goodpasture's syndrome | Vasculitis |
| Henoch-Schonlein purpura | Vasculitis |
| Hypersensitivity vasculitis | Vasculitis |
| Kawasaki's disease | Vasculitis |
| Nodular vasculitis | Vasculitis |
| Polyarteritis nodosa | Vasculitis |
| Polymyalgia rheumatica | Vasculitis |
| Pulmonary vasculitis | Vasculitis |
| Renal arteritis | Vasculitis |
| Renal vasculitis | Vasculitis |
| Retinal vasculitis | Vasculitis |
| Takayasu's arteritis | Vasculitis |
| Thromboangiitis obliterans | Vasculitis |
| Vascular purpura | Vasculitis |
| Vasculitic rash | Vasculitis |
| Vasculitis | Vasculitis |
| Vasculitis necrotising | Vasculitis |
| Vasculitis gastrointestinal | Vasculitis |
| Rheumatoid vasculitis | Vasculitis |
| Urticarial vasculitis | Vasculitis |
| Cogan's syndrome | Vasculitis |
| Palpable purpura | Vasculitis |
| Lupus vasculitis | Vasculitis |
| Microscopic polyangiitis | Vasculitis |
| Pseudovasculitis | Vasculitis |
| Ocular vasculitis | Vasculitis |
| Segmented hyalinising vasculitis | Vasculitis |
| Capillaritis | Vasculitis |
| Henoch-Schonlein purpura nephritis | Vasculitis |
| Langerhans' cell histiocytosis | Vasculitis |
| Type 2 lepra reaction | Vasculitis |
| Haemorrhagic vasculitis | Vasculitis |
| Granulomatosis with polyangiitis | Vasculitis |
| Chronic pigmented purpura | Vasculitis |
| Radiation vasculitis | Vasculitis |
| Diabetic arteritis | Vasculitis |
| Eosinophilic granulomatosis with polyangiitis | Vasculitis |
| MAGIC syndrome | Vasculitis |
| Central nervous system vasculitis | Vasculitis |
| Anti-glomerular basement membrane disease | Vasculitis |
| Retinal occlusive vasculitis | Vasculitis |
| Haemorrhagic occlusive retinal vasculitis | Vasculitis |
| Immune-mediated vasculitis | Vasculitis |
| Patellofemoral pain syndrome | Arthritis |
| Addison's disease | Other immune-mediated disorders |
| Amyloidosis | Other immune-mediated disorders |
| Antiphospholipid syndrome | Other immune-mediated disorders |
| Autoimmune hepatitis | Other immune-mediated disorders |
| Cardiac amyloidosis | Other immune-mediated disorders |
| Cardiac sarcoidosis | Other immune-mediated disorders |
| Cholangitis sclerosing | Other immune-mediated disorders |
| Coeliac disease | Other immune-mediated disorders |
| Cold type haemolytic anaemia | Other immune-mediated disorders |
| Colitis ulcerative | Other immune-mediated disorders |
| Collagen-vascular disease | Other immune-mediated disorders |
| Concentric sclerosis | Other immune-mediated disorders |
| CREST syndrome | Other immune-mediated disorders |
| Crohn's disease | Other immune-mediated disorders |
| Cutaneous amyloidosis | Other immune-mediated disorders |
| Cutaneous sarcoidosis | Other immune-mediated disorders |
| Cystitis interstitial | Other immune-mediated disorders |
| Dermatomyositis | Other immune-mediated disorders |
| Dressler's syndrome | Other immune-mediated disorders |
| Encephalitis post immunisation | Other immune-mediated disorders |
| Gastritis | Other immune-mediated disorders |
| Glomerulonephritis | Other immune-mediated disorders |
| Glomerulonephritis rapidly progressive | Other immune-mediated disorders |
| Graves' disease | Other immune-mediated disorders |
| Guillain-Barre syndrome | Other immune-mediated disorders |
| Inflammatory bowel disease | Other immune-mediated disorders |
| Insulin autoimmune syndrome | Other immune-mediated disorders |
| Linear IgA disease | Other immune-mediated disorders |
| Lupoid hepatic cirrhosis | Other immune-mediated disorders |
| Lupus encephalitis | Systemic lupus erythematosus |
| Lupus nephritis | Systemic lupus erythematosus |
| Mixed connective tissue disease | Other immune-mediated disorders |
| Morphoea | Other immune-mediated disorders |
| Multiple sclerosis | Other immune-mediated disorders |
| Muscular sarcoidosis | Other immune-mediated disorders |
| Myasthenia gravis | Other immune-mediated disorders |
| Myasthenia gravis neonatal | Other immune-mediated disorders |
| Myasthenic syndrome | Other immune-mediated disorders |
| Myelitis transverse | Other immune-mediated disorders |
| Pernicious anaemia | Other immune-mediated disorders |
| Polyglandular autoimmune syndrome type I | Other immune-mediated disorders |
| Polyglandular autoimmune syndrome type II | Other immune-mediated disorders |
| Autoimmune neutropenia | Other immune-mediated disorders |
| Primary amyloidosis | Other immune-mediated disorders |
| Proctitis ulcerative | Other immune-mediated disorders |
| Psoriasis | Other immune-mediated disorders |
| Pulmonary sarcoidosis | Other immune-mediated disorders |
| Renal amyloidosis | Other immune-mediated disorders |
| Retroperitoneal fibrosis | Other immune-mediated disorders |
| Sarcoidosis | Other immune-mediated disorders |
| Scleroderma | Other immune-mediated disorders |
| Secondary amyloidosis | Other immune-mediated disorders |
| Sjogren's syndrome | Other immune-mediated disorders |
| Systemic lupus erythematosus | Systemic lupus erythematosus |
| Terminal ileitis | Other immune-mediated disorders |
| Thrombocytopenic purpura | Other immune-mediated disorders |
| Vitiligo | Other immune-mediated disorders |
| Leukoencephalomyelitis | Other immune-mediated disorders |
| Autoimmune thyroiditis | Other immune-mediated disorders |
| Ocular myasthenia | Other immune-mediated disorders |
| Lupus-like syndrome | Systemic lupus erythematosus |
| Anti-neutrophil cytoplasmic antibody positive vasculitis | Vasculitis |
| Toxic oil syndrome | Other immune-mediated disorders |
| SAPHO syndrome | Other immune-mediated disorders |
| Cytokine release syndrome | Other immune-mediated disorders |
| POEMS syndrome | Other immune-mediated disorders |
| Evans syndrome | Other immune-mediated disorders |
| Cutaneous lupus erythematosus | Systemic lupus erythematosus |
| Lupus pneumonitis | Systemic lupus erythematosus |
| Chronic inflammatory demyelinating polyradiculoneuropathy | Other immune-mediated disorders |
| Neonatal lupus erythematosus | Systemic lupus erythematosus |
| Subacute cutaneous lupus erythematosus | Systemic lupus erythematosus |
| Acute cutaneous lupus erythematosus | Systemic lupus erythematosus |
| Chronic cutaneous lupus erythematosus | Systemic lupus erythematosus |
| Pericarditis lupus | Systemic lupus erythematosus |
| Lupus endocarditis | Systemic lupus erythematosus |
| Gastrointestinal amyloidosis | Other immune-mediated disorders |
| Postpericardiotomy syndrome | Other immune-mediated disorders |
| Endocrine ophthalmopathy | Other immune-mediated disorders |
| Immune-mediated pancytopenia | Other immune-mediated disorders |
| Demyelinating polyneuropathy | Other immune-mediated disorders |
| Peritonitis lupus | Systemic lupus erythematosus |
| Relapsing-remitting multiple sclerosis | Other immune-mediated disorders |
| Secondary progressive multiple sclerosis | Other immune-mediated disorders |
| Primary progressive multiple sclerosis | Other immune-mediated disorders |
| Loefgren syndrome | Other immune-mediated disorders |
| Neuropsychiatric lupus | Systemic lupus erythematosus |
| Lymphocytic hypophysitis | Other immune-mediated disorders |
| Polyglandular autoimmune syndrome type III | Other immune-mediated disorders |
| Eosinophilic oesophagitis | Other immune-mediated disorders |
| Autoimmune myocarditis | Other immune-mediated disorders |
| Dialysis amyloidosis | Other immune-mediated disorders |
| Polychondritis | Other immune-mediated disorders |
| Multifocal motor neuropathy | Other immune-mediated disorders |
| Lewis-Sumner syndrome | Other immune-mediated disorders |
| Ocular sarcoidosis | Other immune-mediated disorders |
| Tongue amyloidosis | Other immune-mediated disorders |
| Autoimmune inner ear disease | Other immune-mediated disorders |
| Lupus myocarditis | Systemic lupus erythematosus |
| Inclusion body myositis | Other immune-mediated disorders |
| Progressive relapsing multiple sclerosis | Other immune-mediated disorders |
| Marburg's variant multiple sclerosis | Other immune-mediated disorders |
| Type 1 diabetes mellitus | Other immune-mediated disorders |
| Lupus hepatitis | Systemic lupus erythematosus |
| Lupus enteritis | Systemic lupus erythematosus |
| Shrinking lung syndrome | Systemic lupus erythematosus |
| Lupus pancreatitis | Systemic lupus erythematosus |
| Ocular pemphigoid | Other immune-mediated disorders |
| Interstitial granulomatous dermatitis | Other immune-mediated disorders |
| Cerebral amyloid angiopathy | Other immune-mediated disorders |
| Fibrillary glomerulonephritis | Other immune-mediated disorders |
| Pulmonary renal syndrome | Other immune-mediated disorders |
| Liver sarcoidosis | Other immune-mediated disorders |
| Overlap syndrome | Other immune-mediated disorders |
| Antisynthetase syndrome | Other immune-mediated disorders |
| Palisaded neutrophilic granulomatous dermatitis | Other immune-mediated disorders |
| Marine Lenhart syndrome | Other immune-mediated disorders |
| Autoimmune pancreatitis | Other immune-mediated disorders |
| Tubulointerstitial nephritis and uveitis syndrome | Other immune-mediated disorders |
| Hashimoto's encephalopathy | Other immune-mediated disorders |
| Autoimmune lymphoproliferative syndrome | Other immune-mediated disorders |
| Autoimmune neuropathy | Other immune-mediated disorders |
| Satoyoshi syndrome | Other immune-mediated disorders |
| Metastatic cutaneous Crohn's disease | Other immune-mediated disorders |
| Reynold's syndrome | Other immune-mediated disorders |
| Posterior reversible encephalopathy syndrome | Other immune-mediated disorders |
| Clinically isolated syndrome | Other immune-mediated disorders |
| Rasmussen encephalitis | Other immune-mediated disorders |
| Chronic recurrent multifocal osteomyelitis | Other immune-mediated disorders |
| Susac's syndrome | Other immune-mediated disorders |
| Undifferentiated connective tissue disease | Other immune-mediated disorders |
| Autoimmune aplastic anaemia | Other immune-mediated disorders |
| Autoimmune retinopathy | Other immune-mediated disorders |
| Noninfectious myelitis | Other immune-mediated disorders |
| Stiff person syndrome | Other immune-mediated disorders |
| Autoinflammatory disease | Other immune-mediated disorders |
| Encephalitis autoimmune | Other immune-mediated disorders |
| Birdshot chorioretinopathy | Other immune-mediated disorders |
| Chronic autoimmune glomerulonephritis | Other immune-mediated disorders |
| Autoimmune haemolytic anaemia | Systemic lupus erythematosus |
| Pyostomatitis vegetans | Other immune-mediated disorders |
| Stoma site vasculitis | Other immune-mediated disorders |
| Noninfective encephalitis | Other immune-mediated disorders |
| Noninfective encephalomyelitis | Other immune-mediated disorders |
| Lupus cystitis | Systemic lupus erythematosus |
| Granulomatous dermatitis | Other immune-mediated disorders |
| Morvan syndrome | Other immune-mediated disorders |
| Hepatic amyloidosis | Other immune-mediated disorders |
| Autoimmune demyelinating disease | Other immune-mediated disorders |
| Autoimmune dermatitis | Other immune-mediated disorders |
| Autoimmune uveitis | Other immune-mediated disorders |
| Autoimmune encephalopathy | Other immune-mediated disorders |
| Autoimmune colitis | Other immune-mediated disorders |
| Central nervous system lupus | Systemic lupus erythematosus |
| Autoimmune hypothyroidism | Other immune-mediated disorders |
| Acute motor-sensory axonal neuropathy | Other immune-mediated disorders |
| Acute motor axonal neuropathy | Other immune-mediated disorders |
| Juvenile polymyositis | Other immune-mediated disorders |
| Bickerstaff's encephalitis | Other immune-mediated disorders |
| Autoimmune nephritis | Systemic lupus erythematosus |
| IgM nephropathy | Systemic lupus erythematosus |
| Immunoglobulin G4 related disease | Other immune-mediated disorders |
| C3 glomerulopathy | Other immune-mediated disorders |
| Neurosarcoidosis | Other immune-mediated disorders |
| Anti-myelin-associated glycoprotein associated polyneuropathy | Other immune-mediated disorders |
| Tumefactive multiple sclerosis | Other immune-mediated disorders |
| Systemic scleroderma | Other immune-mediated disorders |
| Immune-mediated enterocolitis | Other immune-mediated disorders |
| Immune-mediated hepatitis | Other immune-mediated disorders |
| Immune-mediated neuropathy | Other immune-mediated disorders |
| Immune-mediated endocrinopathy | Other immune-mediated disorders |
| Autoimmune pericarditis | Systemic lupus erythematosus |
| Radiologically isolated syndrome | Other immune-mediated disorders |
| Stiff leg syndrome | Other immune-mediated disorders |
| Rheumatic brain disease | Other immune-mediated disorders |
| Lupus myositis | Systemic lupus erythematosus |
| Autoimmune anaemia | Systemic lupus erythematosus |
| Neuromyelitis optica pseudo relapse | Other immune-mediated disorders |
| Primary biliary cholangitis | Other immune-mediated disorders |
| Alloimmune hepatitis | Other immune-mediated disorders |
| IPEX syndrome | Other immune-mediated disorders |
| Relapsing multiple sclerosis | Other immune-mediated disorders |
| Autoimmune lung disease | Other immune-mediated disorders |
| Immune thrombocytopenia | Systemic lupus erythematosus |
| Autoimmune enteropathy | Other immune-mediated disorders |
| C1q nephropathy | Other immune-mediated disorders |
| Subacute inflammatory demyelinating polyneuropathy | Other immune-mediated disorders |
| Vogt-Koyanagi-Harada disease | Other immune-mediated disorders |
| Autoimmune myositis | Other immune-mediated disorders |
| Immune-mediated myocarditis | Other immune-mediated disorders |
| Immune-mediated uveitis | Other immune-mediated disorders |
| Immune-mediated nephritis | Systemic lupus erythematosus |
| Immune-mediated thyroiditis | Other immune-mediated disorders |
| Immune-mediated pancreatitis | Other immune-mediated disorders |
| Immune-mediated myositis | Other immune-mediated disorders |
| Immune-mediated encephalitis | Other immune-mediated disorders |
| Immune-mediated hypothyroidism | Other immune-mediated disorders |
| Immune-mediated dermatitis | Systemic lupus erythematosus |
| Immune effector cell-associated neurotoxicity syndrome | Other immune-mediated disorders |
| Immune-mediated cholangitis | Other immune-mediated disorders |
| Immune-mediated hyperthyroidism | Other immune-mediated disorders |
| Autoimmune cholangitis | Other immune-mediated disorders |
| Acquired ATTR amyloidosis | Other immune-mediated disorders |
| Autoimmune blistering disease | Other immune-mediated disorders |
| Neonatal Crohn's disease | Other immune-mediated disorders |
| Immune-mediated gastritis | Other immune-mediated disorders |
| Autoinflammation with infantile enterocolitis | Other immune-mediated disorders |
| Immune-mediated encephalopathy | Other immune-mediated disorders |
| Multisystem inflammatory syndrome in children | Other immune-mediated disorders |
| De novo purine synthesis inhibitors associated acute inflammatory syndrome | Other immune-mediated disorders |
| Immune-mediated hypophysitis | Other immune-mediated disorders |
| Immune-mediated oesophagitis | Other immune-mediated disorders |
| Immune-mediated cystitis | Other immune-mediated disorders |
| Lupus anticoagulant hypoprothrombinaemia syndrome | Systemic lupus erythematosus |
| Immune-mediated lung disease | Other immune-mediated disorders |
| Food protein-induced enterocolitis syndrome | Other immune-mediated disorders |
| Anti-LRP2 nephropathy | Other immune-mediated disorders |
| Rheumatoid arthritis-associated interstitial lung disease | Other immune-mediated disorders |
| Myelin oligodendrocyte glycoprotein antibody-associated disease | Other immune-mediated disorders |
| Multisystem inflammatory syndrome in adults | Other immune-mediated disorders |
| Idiopathic inflammatory myopathy | Other immune-mediated disorders |
| Rheumatoid meningitis | Other immune-mediated disorders |
| Multisystem inflammatory syndrome | Other immune-mediated disorders |
| Acute necrotising myelitis | Other immune-mediated disorders |
| Thrombosis with thrombocytopenia syndrome | Other immune-mediated disorders |
| Chronic inflammatory response syndrome | Other immune-mediated disorders |
| Diffuse infiltrative lymphocytosis syndrome | Other immune-mediated disorders |
| Rheumatoid bursitis | Arthritis |
| Food protein-induced enteropathy | Other immune-mediated disorders |
| ASIA syndrome | Other immune-mediated disorders |
| Sarcoidosis of lymph node | Other immune-mediated disorders |
| Rheumatoid pleuritis | Other immune-mediated disorders |
| Immune-mediated scleritis | Other immune-mediated disorders |
| Immune-mediated pericarditis | Other immune-mediated disorders |
| Food protein-induced allergic proctocolitis | Other immune-mediated disorders |
| Enhanced respiratory disease | Other immune-mediated disorders |
| Immune-mediated polyserositis | Systemic lupus erythematosus |
| CANOMAD syndrome | Other immune-mediated disorders |
| Immune-mediated myelitis | Other immune-mediated disorders |
| Immune-complex membranoproliferative glomerulonephritis | Systemic lupus erythematosus |
| Immune effector cell-associated HLH-like syndrome | Other immune-mediated disorders |
| Immune-mediated cholestasis | Other immune-mediated disorders |
| Immune-mediated cytopenia | Other immune-mediated disorders |
| Autoimmune pancytopenia | Other immune-mediated disorders |
| Testicular autoimmunity | Other immune-mediated disorders |
| Autoimmune hyperlipidaemia | Other immune-mediated disorders |
| IgA nephropathy | Systemic lupus erythematosus |
| Systemic lupus erythematosus rash | Systemic lupus erythematosus |
| Lupus pleurisy | Systemic lupus erythematosus |
| Immune-mediated renal disorder | Systemic lupus erythematosus |
