# Supplementary material for: Disproportionality analysis of European safety reports on autoimmune and rheumatic diseases following COVID-19 vaccination
Source: Sci Rep. 2025 Apr 27;15:14740. doi: 10.1038/s41598-025-98313-4 (PMC12034749; doi:10.1038/s41598-025-98313-4)
Supplement: Supplementary file 2 — Supplementary Material 2 [file 41598_2025_98313_MOESM2_ESM.docx]

**Table S2.** List of drugs (5^th^ anatomical therapeutic chemical level) included in the analysis to identify the individual case safety reports pertaining to patients with pre-existing rheumatic diseases, grouped by their 4^th^, 3^rd^, and 2^nd^ anatomical therapeutic chemical levels.

| **ATC (2^nd^ level)** | **ATC (3^rd^ level)** | **ATC (4^th^ level)** | **ATC (5^th^ level)** |
| --- | --- | --- | --- |
| M01 - Antinflammatory and Antirheumatic products | M01C - Specific Antirheumatic Agents | M01CC - Penicillamine and Similar Agents | BUCILLAMINE |
|  |  |  | PENICILLAMINE |
|  |  | M01CB - Gold Preparations | AUROTIOPROL |
|  |  |  | AUROTHIOGLUCOSE |
|  |  |  | AURANOFIN |
|  |  |  | SODIUM AUROTIOSULFATE |
|  |  |  | SODIUM AUROTHIOMALATE |
|  |  | M01CA - Quinolines | OXYCINCHOPHEN |
|  | M01B - Antinflammatory/antirheumatic agents in combination | M01BA - Antinflammatory/antirheumatic agents in combination with corticosteroids | ACETYLSALICYLIC ACID |
|  |  |  | DIPYROCETYL |
|  |  |  | PHENYLBUTAZONE |
|  | M01A- Antinflammatory and antirheumatic products | M01AA - Butylpirazolidines | KEBUZONE |
|  |  |  | MOFEBUTAZONE |
|  |  |  | OXYPHENBUTAZONE |
|  |  |  | RABEPRAZOLE |
|  |  | M01AB - Acectic acid derivates and relates substances | ACECLOFENAC |
|  |  |  | ACEMETACIN |
|  |  |  | ALCLOFENAC |
|  |  |  | BUFEXAMAC |
|  |  |  | BUMADIZONE |
|  |  |  | DICLOFENAC |
|  |  |  | DIFENPIRAMIDE |
|  |  |  | ETODOLAC |
|  |  |  | FENTIAZAC |
|  |  |  | INDOMETHACIN |
|  |  |  | KETOROLAC |
|  |  |  | LONAZOLAC |
|  |  |  | OXAMETACIN |
|  |  |  | PROGLUMETACIN |
|  |  |  | SULINDAC |
|  |  |  | TOLMETIN |
|  |  |  | ZOMEPIRAC |
|  |  | M01AC - Oxicams | DROXICAM |
|  |  |  | LORNOXICAM |
|  |  |  | MELOXICAM |
|  |  |  | PIROXICAM |
|  |  |  | TENOXICAM |
|  |  | M01AE - Propionic Acid derivates | MISOPROSTOL |
|  |  |  | NAPROXEN |
|  |  |  | KETOPROFEN |
|  |  |  | ESOMEPRAZOLE |
|  |  |  | IBUPROFEN |
|  |  |  | NAPROXCINOD |
|  |  |  | DEXKETOPROFEN |
|  |  |  | ALMINOPROFEN |
|  |  |  | FLUNOXAPROFEN |
|  |  |  | DEXIBUPROFEN |
|  |  |  | IBUPROXAM |
|  |  |  | OXAPROZIN |
|  |  |  | TIAPROFENIC ACID |
|  |  |  | INDOPROFEN |
|  |  |  | FLURBIPROFEN |
|  |  |  | PIRPROFEN |
|  |  |  | SUPROFEN |
|  |  |  | BENOXAPROFEN |
|  |  |  | FENBUFEN |
|  |  |  | FENOPROFEN |
|  |  | M01AG - Fenamates | MECLOFENAMIC ACID |
|  |  |  | FLUFENAMIC ACID |
|  |  |  | TOLFENAMIC ACID |
|  |  |  | MEFENAMIC ACID |
|  |  | M01AH - Coxibs | CELECOXIB |
|  |  |  | ETORICOXIB |
|  |  |  | LUMIRACOXIB |
|  |  |  | PARECOXIB |
|  |  |  | POLMACOXIB |
|  |  |  | ROFECOXIB |
|  |  |  | VALDECOXIB |
|  |  | M01AX - Other antiinflammatory and antirheumatic agents, non steroids | FEPRAZONE |
|  |  |  | CHONDROITIN SULFATE |
|  |  |  | OXACEPROL |
|  |  |  | TENIDAP |
|  |  |  | MORNIFLUMATE |
|  |  |  | DIACEREIN |
|  |  |  | NIMESULIDE |
|  |  |  | ORGOTEIN |
|  |  |  | PROQUAZONE |
|  |  |  | BENZYDAMINE |
|  |  |  | GLUCOSAMINE |
|  |  |  | AZAPROPAZONE |
|  |  |  | NIFLUMIC ACID |
|  |  |  | NABUMETONE |
