# Supplementary material for: Disproportionality analysis of European safety reports on autoimmune and rheumatic diseases following COVID-19 vaccination
Source: Sci Rep. 2025 Apr 27;15:14740. doi: 10.1038/s41598-025-98313-4 (PMC12034749; doi:10.1038/s41598-025-98313-4)
Supplement: Supplementary file 3 — Supplementary Material 3 [file 41598_2025_98313_MOESM3_ESM.docx]

**Table S3.** Disproportionality analysis including adverse events reported by healthcare professionals grouped by each Standardized MedDRA Query (SMQ).

| **SMQ** | **Vaccine** | **Comparator** | **Chi-squared** | **ROR (95% CI)** |
| --- | --- | --- | --- | --- |
| **Arthritis** | Tozinameran | ChAd | 33.69 | 1.24 (1.15-1.34) |
|  |  | Elasomeran | 3.06 | 1.08 (0.99-1.17) |
|  |  | Ad26.Cov2.S | 52.62 | 1.81 (1.54-2.13) |
|  |  | Mix | 2.12 | 0.81 (0.63-1.07) |
|  | ChAd | Elasomeran | 9.23 | 0.86 (0.79-0.95) |
|  |  | Ad26.Cov2.S | 19.06 | 1.45 (1.23-1.73) |
|  |  | Mix | 9.52 | 0.66 (0.50-0.86) |
|  | Elasomeran | Ad26.Cov2.S | 35.5 | 1.68 (1.41-2.00) |
|  |  | Mix | 3.87 | 0.76 (0.58-1.00) |
|  | Ad26.Cov2.S | Mix | **27.15** | **0.45 (0.33-0.62)** |
| **Vasculitis** | Tozinameran | ChAd | 43.76 | 1.33 (1.22-1.45) |
|  |  | Elasomeran | 33.11 | 1.33 (1.21-1.47) |
|  |  | Ad26.Cov2.S | **66.91** | **2.33 (1.89-2.90)** |
|  |  | Mix | 2.98 | 1.36 (0.97-1.96) |
|  | ChAd | Elasomeran | < 0.01 | 0.99 (0.89-1.12) |
|  |  | Ad26.Cov2.S | 26.21 | 1.75 (1.41-2.19) |
|  |  | Mix | < 0.01 | 1.02 (0.72-1.47) |
|  | Elasomeran | Ad26.Cov2.S | 25.01 | 1.75 (1.40-2.20) |
|  |  | Mix | < 0.01 | 1.02 (0.72-1.48) |
|  | Ad26.Cov2.S | Mix | 6.83 | 0.58 (0.39-0.88) |
| **Tendinopathies** | Tozinameran | ChAd | 23.1 | 1.50 (1.27-1.77) |
|  |  | Elasomeran | 12.33 | 1.39 (1.16-1.69) |
|  |  | Ad26.Cov2.S | **25.89** | **3.25 (2.02-5.54)** |
|  |  | Mix | 0.33 | 1.25 (0.68-2.55) |
|  | ChAd | Elasomeran | 0.34 | 0.93 (0.75-1.16) |
|  |  | Ad26.Cov2.S | **9.62** | **2.17 (1.33-3.75)** |
|  |  | Mix | 0.16 | 0.83 (0.45-1.72) |
|  | Elasomeran | Ad26.Cov2.S | **11.26** | **2.33 (1.41-4.05)** |
|  |  | Mix | 0.03 | 0.89 (0.48-1.85) |
|  | Ad26.Cov2.S | **Mix** | **5.34** | **0.38 (0.17-0.91)** |
| **Systemic lupus erythematosus** | Tozinameran | ChAd | 33.98 | 0.75 (0.68-0.83) |
|  |  | Elasomeran | 23.59 | 0.76 (0.68-0.85) |
|  |  | Ad26.Cov2.S | 0.12 | 0.96 (0.79-1.18) |
|  |  | Mix | 0.89 | 1.26 (0.81-2.02) |
|  | ChAd | Elasomeran | 0.06 | 1.01 (0.90-1.14) |
|  |  | Ad26.Cov2.S | 5.51 | 1.28 (1.04-1.58) |
|  |  | Mix | 5.14 | 1.67 (1.08-2.70) |
|  | Elasomeran | Ad26.Cov2.S | 4.49 | 1.26 (1.02-1.56) |
|  |  | Mix | 4.73 | 1.65 (1.06-2.67) |
|  | Ad26.Cov2.S | Mix | 1.05 | 1.30 (0.81-2.18) |
| **Other immune-mediated disorders** | Tozinameran | ChAd | 68.12 | 0.77 (0.72-0.82) |
|  |  | Elasomeran | 18.86 | 0.85 (0.80-0.92) |
|  |  | Ad26.Cov2.S | **207.41** | **0.41 (0.36-0.47)** |
|  |  | Mix | 0.87 | 0.89 (0.70-1.13) |
|  | ChAd | Elasomeran | 6.75 | 1.11 (1.02-1.20) |
|  |  | Ad26.Cov2.S | 93.62 | 0.53 (0.47-0.60) |
|  |  | Mix | 1.22 | 1.15 (0.90-1.47) |
|  | Elasomeran | Ad26.Cov2.S | **119.37** | **0.48 (0.42-0.55)** |
|  |  | Mix | 0.06 | 1.04 (0.81-1.33) |
|  | Ad26.Cov2.S | Mix | **33.58** | **2.16 (1.65-2.82)** |
